# Acute Acalculous Cholecystitis Secondary to Acute Pyelonephritis in a Young Woman: A Diagnostic Pitfall in the Emergency Department

**DOI:** 10.7759/cureus.101362

**Published:** 2026-01-12

**Authors:** Michal Balenkowski, Mateusz Antonow, Agnieszka Gorska, Mariusz Sieminski, Katarzyna Golabek

**Affiliations:** 1 Department of Emergency Medicine, Medical University of Gdańsk, Gdańsk, POL; 2 Emergency Department, University Clinical Centre in Gdańsk, Gdańsk, POL

**Keywords:** acute acalculous cholecystitis (aac), emergency service, gallbladder diseases, non-obstructive pyelonephritis, point-of-care ultrasonography

## Abstract

Acute acalculous cholecystitis (AAC) is typically observed in critically ill patients, making its diagnosis in otherwise healthy individuals a significant clinical challenge. We present a rare case of a 29-year-old woman initially suspected of having AAC based on clinical assessment and ultrasonography. While initial imaging fulfilled the sonographic criteria for acalculous cholecystitis, subsequent contrast-enhanced computed tomography revealed right-sided acute pyelonephritis with secondary reactive gallbladder changes. The patient was successfully managed conservatively with intravenous followed by oral antibiotics, resulting in complete recovery without surgical intervention. This case underscores the potential for renal pathology to mimic primary biliary disease and highlights the critical role of advanced imaging in preventing unnecessary invasive procedures in the emergency department.

## Introduction

The gold standard for diagnosing cholecystitis remains ultrasonography. In emergency situations, both point-of-care ultrasonography (POCUS) examinations and urgent ultrasound performed by a radiologist are usually decisive [1-4]. However, in the case of acalculous cholecystitis, caution and diagnostic vigilance are necessary [5-7]. Repeated physical examination during the initial observation period, along with the use of additional diagnostic methods, constitutes a valuable complement to clinical management and helps to avoid unnecessary surgical intervention.

A thorough search of literature (using terms such as "acute acalculous cholecystitis", "pyelonephritis", "reactive gallbladder", and "point-of-care ultrasound") identified studies on acute acalculous cholecystitis (AAC), its radiologic evaluation (including ultrasound and CT), prognostic implications, and selected case reports linking acalculous cholecystitis with other systemic conditions. However, no reports describing AAC secondary to acute pyelonephritis were found. This suggests that the presented constellation and diagnostic challenge are likely rare and add incremental educational value for emergency physicians. This paper presents a case illustrating such a clinical situation.

## Case presentation

A 29-year-old woman was admitted to the emergency department due to severe abdominal pain, nausea, fever, and muscle pain for four days. She correlated her symptoms with the first-time tirzepatide injection in the course of obesity treatment. She was also treated with venlafaxine and chlorprothixene due to depressive disorder. Physical examination revealed tenderness in the right upper quadrant of the abdomen with a positive Blumberg’s sign in this area. No other symptoms were present at the time of admission.

Point-of-care ultrasonography (POCUS) showed thickening of the gallbladder wall and slight enlargement of the right kidney, with no evidence of urine stasis. A formal ultrasound confirmed thickening of the gallbladder wall up to 5 mm with pericholecystic oedema. No deposits or dilatation of the common bile duct or intrahepatic bile ducts were detected. Enlargement of the liver up to 18 cm was observed. The ultrasound image was consistent with the diagnosis of acalculous cholecystitis.

Blood tests showed an increased white blood cell count with neutrophilia, markedly elevated C-reactive protein and procalcitonin levels, mildly elevated creatinine, normal bilirubin, and gamma-glutamyl transpeptidase (Table 1). There was no elevation of other liver enzymes. A urine test revealed increased erythrocytes and leucocytes per high-power field.

**Table 1:** Laboratory findings HPF: high-power field, GGTP: gamma-glutamyl transpeptidase

Parameter	Admission	After 5 days	Reference range
White blood cells	16.31	9.51	4-10x10^9^/L
Neutrophils	11.11	6.02	2-7x10^9^/L
C-reactive protein	301.7	70.3	<5 mg/L
Procalcitonin	43.47	3.04	<0.5 ng/mL
Creatinine	1.23	0.97	0.55-1.02 mg/dL
Bilirubin	0.45	-	0.1-1.2 mg/dL
GGTP	46	-	<38 U/L
Urine erythrocytes	4-10	-	<3 per HPF
Urine leucocytes	19-25	-	<5 per HPF

**Figure 1:**
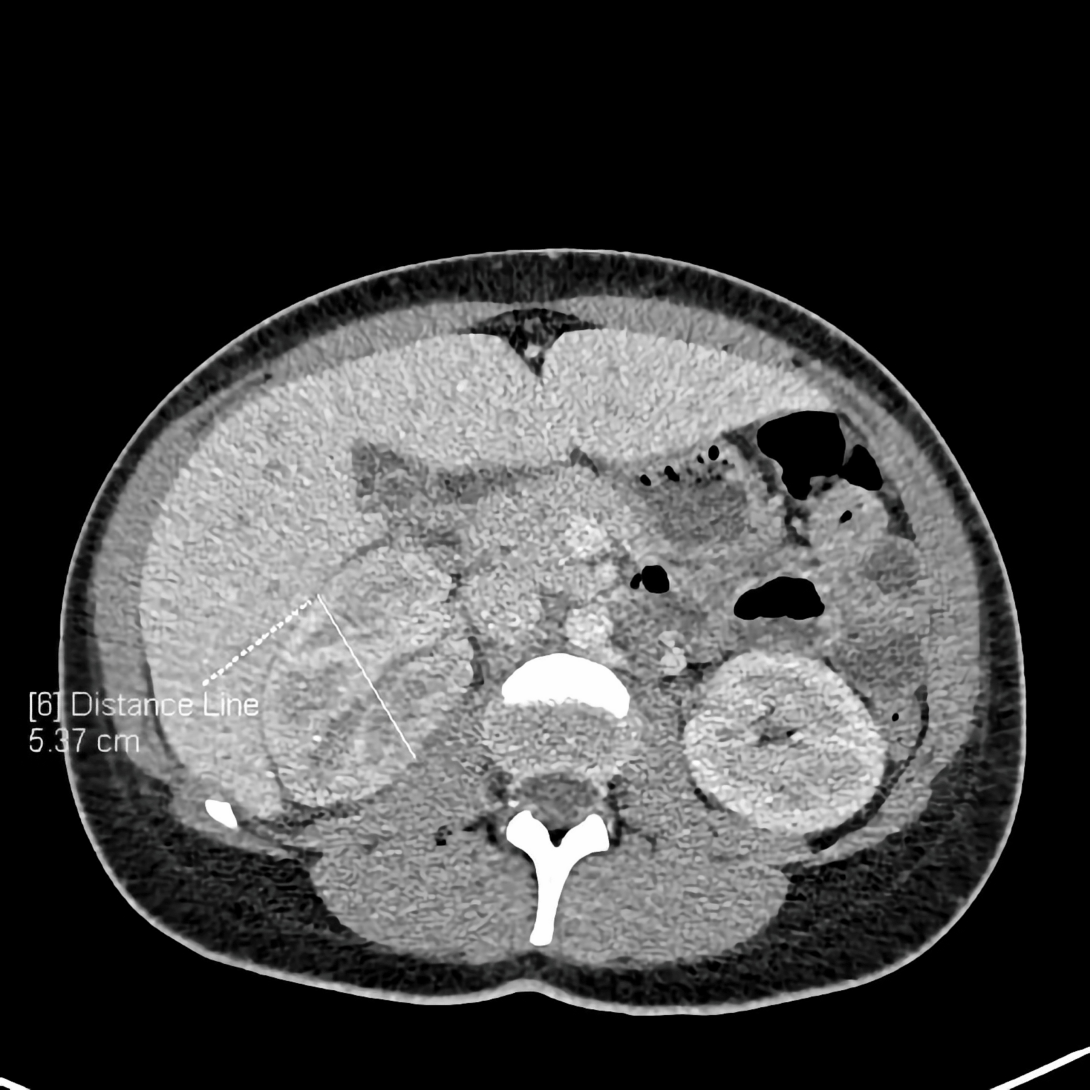
CT scan (axial) showing enlarged right kidney CT: computed tomography

**Figure 2:**
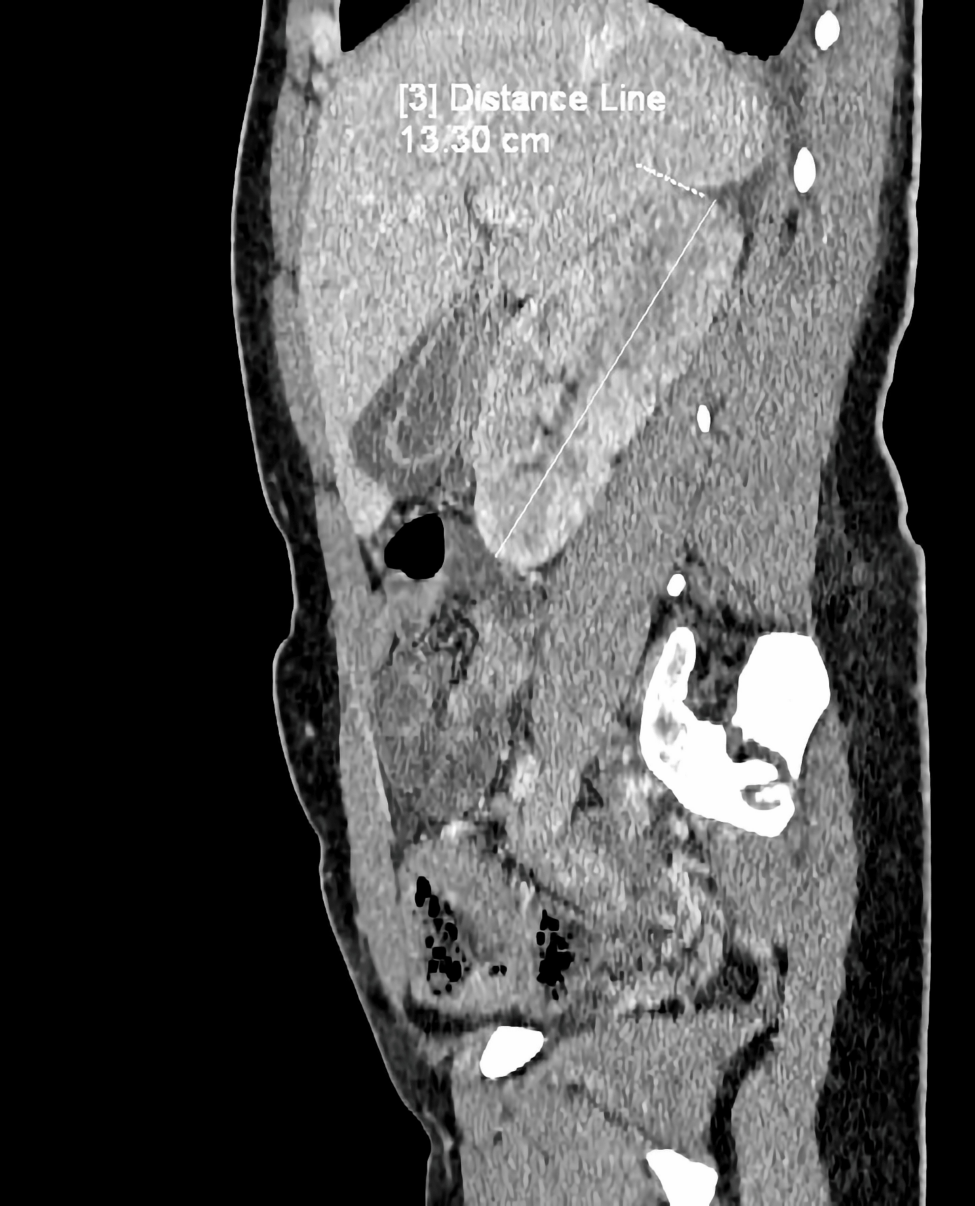
CT scan (sagittal) showing enlarged right kidney and pericholecystic inflammatory changes CT: computed tomography

During examination by a consulting surgeon, about four hours after the initial examination, a positive Goldflam’s sign (costovertebral angle tenderness) on the right side occurred. A CT scan was performed, and the image revealed liver enlargement up to 20 cm, periportal and perivesicular edema, and enlargement of the right kidney to 130 x 54 x 67 mm with numerous wedge-shaped areas of poor contrast enhancement, primarily in the arterial phase (Figures 1-2). Fluid around the kidney and a small amount of fluid in the peritoneal cavity and both pleural cavities were also observed.

Pyelonephritis with reactive cholecystitis was diagnosed. The patient was admitted to the internal medicine department. Conservative treatment was initiated, including broad-spectrum antibiotic therapy (piperacillin and tazobactam), hydration, and correction of electrolyte imbalances. In the following days, a decrease in inflammatory parameters (Table 1) and resolution of clinical symptoms were observed. The patient was discharged from the hospital after five days in improved condition. De-escalation antibiotic therapy (levofloxacin 1 x 500 mg) was continued at home for 10 days. There was no recurrence of symptoms related to either the gallbladder or the kidneys.

## Discussion

Abdominal pain is one of the most common clinical complaints in emergency departments. Acute cholecystitis accounts for between 3% and 11% of those hospitalizations. Fast and accurate diagnosis is essential because the condition requires surgical treatment, and any delay may lead to severe complications. The 2018 Tokyo Guidelines define diagnostic criteria for acute cholecystitis; right upper quadrant pain, Murphy’s sign, fever, elevated inflammatory markers, and radiological symptoms must be present [2-4]. Our patient had typical symptoms and elevated inflammatory markers at the time of admission, with no evidence of other underlying conditions.

At present, ultrasound is the first-line imaging modality for evaluating acute cholecystitis. POCUS can also be of significant value thanks to its availability and rapid assessment [1-4]. Classic ultrasound has about 81% sensitivity and 83% specificity for diagnosing acute cholecystitis. At the same time, computed tomography has about 94% sensitivity but only 59% specificity [4]. CT examination has no proven superiority over ultrasound in diagnosing acute cholecystitis [1,3,7]. However, unexpected findings, as in our case, might not be detected on an emergency ultrasound.

AAC is diagnosed in only 5% to 10% of patients with cholecystitis, with a multifactorial pathophysiology involving bile stasis or ischemia. It occurs mostly in critically ill patients. Although cholecystitis symptoms are similar to those of cholelithiasis, it carries a much more adverse prognosis, with a mortality rate of around 30% that rises rapidly with any delay in diagnosis. Ultrasound is also the examination of choice in this situation; however, sensitivity and specificity range from 30% to 92% and 89% to 100%, respectively [4-8]. Although our patient was not in a high-risk group (young, healthy woman), blood test results, POCUS, and emergency-mode ultrasound performed by a radiologist showed typical findings. That could have led to surgical management and severe complications concerning pyelonephritis, which was undiagnosed at the time.

CT imaging may supplement information about possible reasons, as well as an extended laboratory test profile. In the literature, AAC appeared as a result of traumatic ischemia or pathology of the right hepatic artery, as well as heart failure [9]. It can also occur in cases of viral infections, such as HBV or EBV [10,11]. In our case, hypoperfusion resulted from the pressure of the edematous right kidney and surrounding tissue due to pyelonephritis. A CT scan was performed due to the occurrence of Goldflam’s sign in the re-evaluation conducted a few hours after admission.

## Conclusions

This case report presents a unique clinical situation where AAC occurred secondary to acute pyelonephritis in a young, previously healthy woman. The case demonstrates several important lessons for emergency physicians. First, while POCUS and formal ultrasonography are valuable first-line diagnostic tools in suspected cholecystitis, they may sometimes lead to premature diagnostic closure, especially in acalculous variants where the underlying cause is unexpected. Second, repeated physical examination during the observation period was essential in this case, as the appearance of costovertebral angle tenderness prompted further investigation that revealed the correct diagnosis. Third, CT imaging can help resolve diagnostic uncertainties and identify alternative or coexisting pathologies that may change clinical management from surgical to conservative approaches. Finally, this case shows the importance of maintaining diagnostic vigilance and considering systemic causes even when initial imaging findings appear to support a surgical diagnosis. The favorable outcome with conservative antibiotic therapy confirms the value of a thorough diagnostic workup before definitive intervention, particularly when clinical features do not entirely match the initial imaging impression.

## References

[1] Chae MS, Kravchuk OA (2025). Point of care ultrasound in rapid diagnosis of acute cholangitis and emphysematous cholecystitis: a case report. Int J Emerg Med.

[2] Joyce A, Snelling PJ, Elsayed T, Keijzers G (2024). Point-of-care ultrasound to diagnose acute cholecystitis in the emergency department: a scoping review. Australas J Ultrasound Med.

[3] Lee D, Appel S, Nunes L (2021). CT findings and outcomes of acute cholecystitis: is additional ultrasound necessary?. Abdom Radiol (NY).

[4] Neitzel E, Laskus J, Mueller PR, Kambadakone A, Srinivas-Rao S, vanSonnenberg E (2025). Part 1: Current concepts in radiologic imaging and intervention in acute cholecystitis. J Intensive Care Med.

[5] Morikawa T, Akada M, Shimizu K (2024). Current status and therapeutic strategy of acute acalculous cholecystitis: Japanese nationwide survey in the era of the Tokyo guidelines. J Hepatobiliary Pancreat Sci.

[6] Chen A, Salehi O, Cevik J (2025). Acalculous cholecystitis as an atypical presentation of viral pericarditis: a case report. Am J Case Rep.

[7] Habib MB, Albandak M, Osman MH, Abbarh S, Sawaf B, Alastal Y, Arabi A (2025). Acalculous cholecystitis: the unexpected mask of de novo heart failure. Clin Case Rep.

[8] Sricharan R, Tejaswini HK (2025). A retrospective cohort study to assess the significance of the Tokyo guidelines-2018 for the diagnosis and management of acute cholecystitis. SN Compr Clin Med.

[9] Yamaguchi S, Kitazono K, Kamiyama T, Ohishi M (2023). Hepatomesenteric trunk dissection complicated with acalculous cholecystitis. Intern Med.

[10] Kaya A, Beycan E, Kaya SY, Özdemir G, Zerdali H, Mert A (2024). Acute acalculous cholecystitis due to hepatitis B virus reactivation: a case report and review of the literature. Trop Doct.

[11] Tsiakalos A, Schinas G, Karatzaferis A (2024). Acalculous cholecystitis as a complication of primary Epstein-Barr virus infection: a case-based scoping review of the literature. Viruses.

